# Comparative Brain Morphology of the Greenland and Pacific Sleeper Sharks and its Functional Implications

**DOI:** 10.1038/s41598-019-46225-5

**Published:** 2019-07-11

**Authors:** Kara E. Yopak, Bailey C. McMeans, Christopher G. Mull, Kirk W. Feindel, Kit M. Kovacs, Christian Lydersen, Aaron T. Fisk, Shaun P. Collin

**Affiliations:** 1 Department of Biology and Marine Biology and the UNCW Center for Marine Science, University of North Carolina Wilmington, Wilmington, NC 28403 United States; 2 Department of Biology, University of Toronto Mississauga, Mississauga, ON L5L 1C6 Canada; 3 Earth to Ocean Research Group, Department of Biological Sciences, Simon Fraser University, Burnaby, British Columbia V5A 1S6 Canada; 4 Center for Microscopy Characterisation and Analysis, University of Western Australia, Crawley, WA 6009 Australia; 5 Norwegian Polar Institute, Fram Centre, N-9296 Tromsø, Norway; 6 Great Lakes Institute for Environmental Research, University of Windsor, N9B 3P4 Windsor, ON Canada; 7 Oceans Graduate School and The Oceans Institute, The University of Western Australia, Crawley, WA 6009 Australia; 8 School of Life Sciences, La Trobe University, Bundoora 3086, Victoria, Australia

**Keywords:** Evolution, Neuroscience

## Abstract

In cartilaginous fishes, variability in the size of the brain and its major regions is often associated with primary habitat and/or specific behavior patterns, which may allow for predictions on the relative importance of different sensory modalities. The Greenland (*Somniosus microcephalus*) and Pacific sleeper (*S*. *pacificus*) sharks are the only non-lamnid shark species found in the Arctic and are among the longest living vertebrates ever described. Despite a presumed visual impairment caused by the regular presence of parasitic ocular lesions, coupled with the fact that locomotory muscle power is often depressed at cold temperatures, these sharks remain capable of capturing active prey, including pinnipeds. Using magnetic resonance imaging (MRI), brain organization of *S*. *microcephalus* and *S*. *pacificus* was assessed in the context of up to 117 other cartilaginous fish species, using phylogenetic comparative techniques. Notably, the region of the brain responsible for motor control (cerebellum) is small and lacking foliation, a characteristic not yet described for any other large-bodied (>3 m) shark. Further, the development of the optic tectum is relatively reduced, while olfactory brain regions are among the largest of any shark species described to date, suggestive of an olfactory-mediated rather than a visually-mediated lifestyle.

## Introduction

Cartilaginous fishes (Class Chondrichthyes) are the oldest lineage of jawed vertebrates and occupy a wide diversity of ecological niches, from shallow coral reef habitats within the epipelagic zone, to the deep abyssal habitats within the benthopelagic zone. Neurobiological variability of the peripheral and central nervous system across a wide range of species suggests these systems are under intense selective pressure to function within narrow environmental constraints^1–4^. In particular, the deep-sea represents the largest and one of the most extreme habitats on earth^5,6^, characterized by extremes in temperature, light, pressure, and biomass^7^. The environmental conditions of the deep-sea are known to drive unique adaptations in morphology and physiology, but little is understood about its effects on the nervous system^8–10^.

In recent years, a comparative approach to understanding brain evolution has provided great insights into correlations between complex brains and behavior. Jerison^11^ pioneered the hypothesis that an increase in brain mass conferred a cognitive advantage and proposed the ‘Principle of Proper Mass’, which asserts that the size of a given brain region will reflect, to some degree, specialized function of that brain region. Although Jerison’s predictions remain to be empirically tested to a great degree across any vertebrate group^12^, and more recent work has suggested neuron density may be more informative than brain size^e.g.^^13,14^, comparative analysis of relative brain size (encephalization) and the relative development of various brain nuclei (brain organization) remain the most widely accepted neuroanatomical proxies for functional capability and sensory specialization. Indeed, correlations between brain organization and ecological and behavioral traits have been documented in nearly every vertebrate group, including mammals^15–18^, birds^19–22^, and bony fishes^8,9,23,24^. Although brain development is evolutionarily constrained^25,26^, variability in brain organization in cartilaginous fishes has been similarly associated with primary habitat, life history traits, and/or specific behavior patterns, even in divergent species that share commonalities in ecology^27–30^. At the peripheral level, significant differences in the size, surface area, and density of the peripheral sense organs of cartilaginous fishes have also been correlated with a range of ecological parameters^31–35^. This variability suggests there may be differences in sensitivity, acuity, and/or detection thresholds^36,37^, although the links between various peripheral characteristics and improved function remains uncertain^e.g.^^38,39^.

The Greenland (*Somniosus microcephalus*)^40^ and Pacific sleeper (*S*. *pacificus*)^41^ sharks are two closely related members of Somniosidae (Class: Chondrichthyes, Order: Squaliformes) and are the only non-lamnid predatory shark species to occur in the Arctic^42–45^. Given the extreme latitudes across which these animals are found, many aspects of their behavior, life history, and basic biology are poorly understood in comparison to other species^46,47^. Described as benthopelagic, *Somniosus spp*. often occur under the ice and at depths between 1200 and 2000 m^48,49^ and are among the largest sharks in the world. Reported maximum lengths for *S*. *microcephalus* range up to 7.56 m^41,43^, although this might be an overestimate, given that most reported sizes range from 2.88 m to 5.04 m^e.g.^^50–52^; *S*. *pacificus* reaches total lengths of up to 4 m^52^. Although information on longevity is scarce, these sharks are also the longest-lived vertebrate currently described, with radiocarbon dating of eye lens nuclei suggesting that *S*. *microcephalus* lives as long as 392 ± 120 years^53^.

Among the most distinctive characteristics of *S*. *microcephalus* and *S*. *pacificus* is the presence of ocular lesions, generated by the ectoparasitic copepod *Ommatokoita elongata* attached to the shark’s cornea^54–56^, indicative of some form of visual impairment or even blindness. Despite what is presumed to be a diminished visual capacity and that locomotory muscle power is often depressed at cold temperatures^57^, such that the sleeper sharks are extremely slow swimmers^58,59^, *S*. *microcephalus* and *S*. *pacificus* remain capable of capturing active prey, feeding on a wide range of mid-water and benthic invertebrates and fishes^52,60^. Marine mammal tissue (including pinnipeds) is also frequently reported as a key dietary component in both of these species^52,61–63^. Although whale tissue is believed to be consumed as carrion^62,64–66^, characteristic bite wounds on living and moribund seals have been attributed to either *S*. *microcephalus* or *S*. *pacificus*, suggesting that both species prey actively on live pinnipeds^52,62,67^. It is assumed that the sharks may capture seals as they enter the water at ice holes^46^ or target sleeping seals^68^; however, it is currently unknown how they localize their prey.

Similar patterns of brain organization within taxa that share ecological or behavioral characteristics suggests that central nervous system morphology may provide insights into sensory specialization. Neural development in other deep-sea sharks reflects their unique environment, with a reduction in the relative size of visually-associated brain regions (i.e., optic tectum)^69^, and an enlargement of regions associated with olfaction (olfactory bulbs)^29^ and the octavolateralis senses (dorsal and medial octavolateralis nuclei)^10,70^, suggestive of a greater reliance on non-visual senses. However, few studies have quantified encephalization or the relative development of major brain areas within the Somniosidae^10^, despite their unique longevity, wide depth distribution, and unresolved predation strategies. Previous studies have suggested that *S*. *microcephalus* has a small brain^71^, with a well-developed olfactory system^72,73^. However, no study to date has examined the relative organization of all major brain regions in these sharks and contextualized it within a comparative framework.

Given the comparative neural dataset that now exists for cartilaginous fishes^3,30,74,75^, the aim of this study is to assess brain size and brain organization (Table 1) of *S*. *microcephalus* and *S*. *pacificus* within the context of a broad range of other species (n = 117), using phylogenetic comparative techniques. Due to the rarity of these samples, magnetic resonance imaging (MRI) was employed, which facilitates the acquisition of high-resolution 3D data of soft tissue structures. This study marks the first effort to gain an evolutionary perspective into the brain of *Somniosus* spp. in comparison to species that represent broad taxonomic and ecological diversity. Although not a functional analysis, assessment of the brain may provide insights into the relative importance of different sensory modalities in these unique shark species.

**Table 1:** Absolute body mass, brain mass, and brain structure mass together with brain size (R_br_) and structure (R_structure_) residual values for the *Somniosus microcephalus* and *S*. *pacificus* specimens examined in this study. Residual values were calculated from pGLS models for brain size (n = 117) and brain structure size (n = 84) (note: data on tectum and tegmentum were only available for 69 species). Standard deviations (±SD) provided where three or more specimens were available.

Brain Morphometrics		Species	
		*Somniosus microcephalus*	*Somniosus pacificus*
Body size	mass (kg) ± SD	347.67 ± 15.5	25.59*
Brain Size	mass (g) ± SD	12.64 ± 0.77	8.153
	residual (R_Br_)	−1.247	1.863
Olfactory Bulbs (OB)	mass (g) ± SD	4.147 ± 0.88	2.536
	residual (R_OB_)	2.955	1.737
Telencephalon (Tel)	mass (g) ± SD	2.131 ± 0.40	1.260
	residual (R_T_)	−3.591	−1.989
Diencephalon (Di)	mass (g) ± SD	0.970 ± 0.08	0.389
	residual (R_D_)	0.391	−0.026
Optic Tectum (OT)	mass (g) ± SD	0.317 ± 0.03	0.198
	residual (R_OT_)	−0.04	−0.066
Tegmentum (Tg)	mass (g) ± SD	0.392 ± 0.03	0.367
	residual (R_Tg_)	−0.340	−0.215
Cerebellum (Cer)	mass (g) ± SD	1.611 ± 0.18	0.956
	residual (R_C_)	−0.607	−0.473
	Foliation Index	1	1
Medulla (Md)	mass (g) ± SD	3.075 ± 0.23	2.449
	residual (R_M_)	1.213	0.997

*Body mass was estimated based on a length-weight relationship^47^.

## Results

### Gross morphology

**Figure 1:**
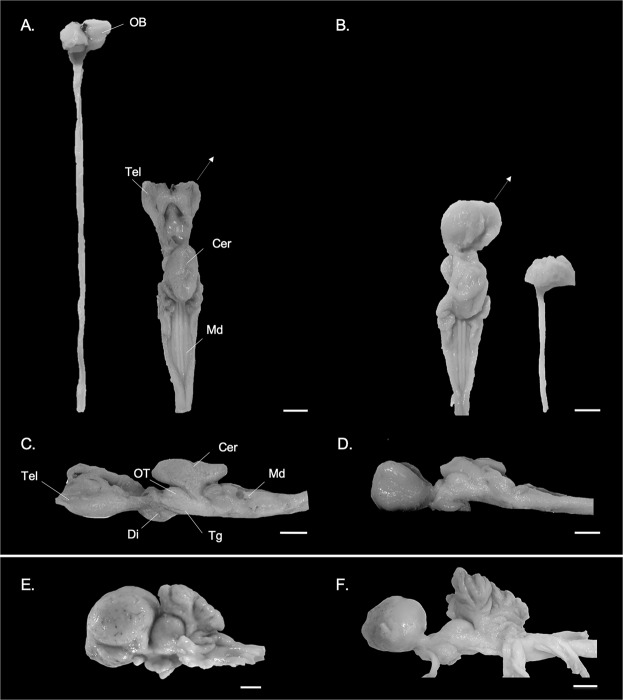
Brain images of a representative specimen of the (**A**,**C**) Greenland shark (*Somniosus microcephalus*) and the (**B**,**D**) Pacific sleeper shark (*S*. *pacificus*) in (**A**,**B**) dorsal and (**C**,**D**) lateral views. (**A,B**) show photomicrographs of one of two paired olfactory bulbs (OB) and the peduncle (olfactory tract), detached from the telencephalon (Tel) at the level anterior to the lateral pallium (dotted arrow), and the remaining brain (telencephalon (Tel), diencephalon (Di), optic tectum (OT), tegmentum (Tg), cerebellum (Cer), and medulla oblongata (Md)). For comparison, photomicrographs of the brain of the (**E**) silky shark *Carcharhinus falciformis* (Photo: T. Lisney) and (**F**) whale shark, *Rhincodon typus* (Photo: K. Yopak) in lateral view are presented. Olfactory bulbs not shown in (**C–F**). Scale bars correspond to 1 cm.

**Figure 2:**
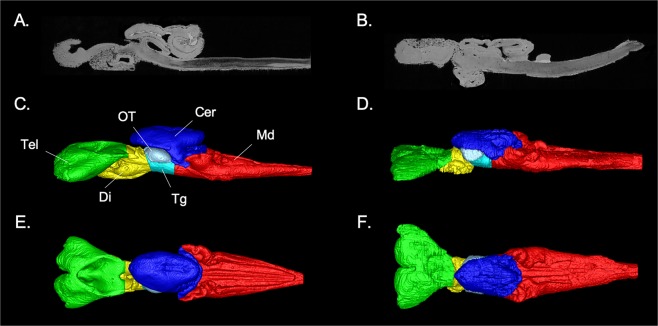
MR images of the brain of the (**A,C,E**) Greenland shark (*Somniosus microcephalus*) and the (**B,D,F**) Pacific sleeper shark (*S*. *pacificus*), including a (**A,B**) sagittal slice of the brain of both species and (**E–F**) digital segmentation of the major structures of the brain in lateral (**C,D**) and dorsal (**E,F**) views. Key: green = telencephalon (Tel), yellow = diencephalon (Di), grey = optic tectum (OT), cyan = tegmentum (Tg), blue = cerebellum (Cer), red = medulla oblongata (Md).

In both *Somniosus microcephalus* and *S*. *pacificus*, the brain (Table 1) occupies only a small proportion (<30%) of the endocranial cavity, although this is most pronounced in *S*. *microcephalus*. The brain of both of these shark species is long and dorsoventrally flattened (Fig. 1A–D), as in other squaliforms. A pair of long olfactory tracts (or peduncles) extend laterally from the telencephalon and terminate in the olfactory bulbs (OBs), which are attached peripherally to large olfactory rosettes. The OBs are divided into medial and lateral hemi-bulbs (Fig. 1A,B). The telencephalon (Fig. 1A–D), which consists of paired evaginated cerebral hemispheres, is similar to other squaliforms^30,75^. It is small in size, with a reduced dorsoposterior region, particularly the central nucleus of the dorsal pallium, in comparison to carcharhinid species, such as the silky shark (*Carcharhinus falciformes*, Carcharhiniformes) (Fig. 1IE). MRI data (Fig. 2) also reveals large telencephalic ventricles in the sleeper sharks, characteristic of squalomorph sharks^76^.

The optic tectum is comprised of two distinct bilateral lobes, where each hemisphere appears small in lateral view (Fig. 1C,D). The cerebellum consists of a central unpaired corpus and laterally situated auricles, where the corpus is small and lacks convolution. It is composed of rostral and caudal lobes, with only a very shallow central sulcus, in comparison to highly foliated species, such as the whale shark (*Rhincodon typus*) (Fig. 1F). The cerebellar auricles are continuous with the acousticolateralis area of the medulla (the dorsal and medial octavolateralis nuclei), which are moderately developed (Fig. 1A–D). Overall, the medulla is wide and open dorsally, a condition typical of most squaliforms and some lamniforms^30,75^.

### Encephalization

**Figure 3:**
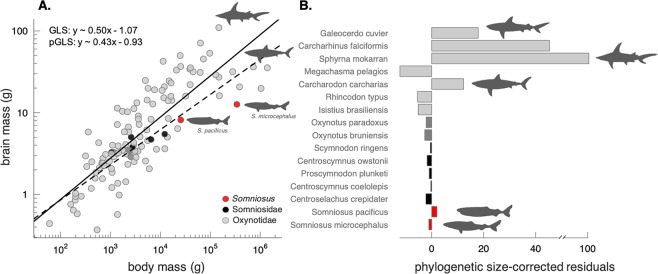
(**A**) Regressions of brain mass with body mass across 117 species of cartilaginous fishes, showing the position of *Somniosus microcephalus* and *S*. *pacificus* (red circles), in comparison to seven other members of the Somniosidae (black circles), Oxynotidae (dark grey circles), and other species of sharks and holocephalans (light grey circles). Both Generalized least squares (GLS - solid line) and pGLS models (hatched line) are shown, with full model results shown in Table 2. (**B**) Phylogenetically size-corrected residuals, which range from R_br_ = −12.37 to R_br_ = 100.31 across 117 species, are presented for *S*. *microcephalus* and *S*. *pacificus* alongside five somniosids, two oxynotids, and *Galeocerdo cuvier*, *Carcharhinus falciformis*, *Sphyrna mokarran*, *Megachasma pelagios*, *Carcharodon carcharias*, *Rhincodon typus*, *Istitius brasiliensis* for broad comparison.

The brain scales with negative allometry against body mass across cartilaginous fishes, as assessed using pGLS (α = 0.43, r^2^ = 0.69, p < 0.001, n = 117) regression (Fig. 3B, Table 2). Phylogenetically-corrected brain size residuals (R_br_) suggest that *S*. *microcephalus* and *S*. *pacificus* have a relatively low degree of encephalization (Fig. 3B). Phylogenetically corrected R_br_ values range from −12.17 (*Megachasma pelagios*) to 100.43 (*Sphyrna mokarran*) (Fig. 3B). *S*. *microcephalus* shows a reduced brain size (GLM, R_br_ = −0.60), which is most pronounced when phylogenetically corrected (pGLS, R_br_ = −1.54), while *S*. *pacificus* has a relative brain size similar to other deep-sea cartilaginous fishes (pGLS, R_br_ = 1.98).

**Table 2:** Generalized least-squares (GLS) and phylogenetic generalized least-squares (pGLS) model results and summary for brain size and brain region size.

Model	Framework	Intercept	Slope	d.f.	F-stat	r^2^	λ
**Brain Size**							
M_brain_ ~ M_body_	GLM	−1.07	0.5	120	261.2	0.68	
	pGLS	−0.93	0.43	120	266.0	0.69	0.86
**Regional Sizes**							
M_Olfactory Bulb_ ~ M_Brain_	GLM	−0.95	0.9	85	390.2	0.82	
	pGLS	−0.98	0.96	85	352.2	0.81	0.54
M_Telencephalon_ ~ M_Brain_	GLM	−0.58	1.14	86	1557	0.95	
	pGLS	−0.58	1.05	86	2392	0.96	0.96
M_Diencephalon_ ~ M_Brain_	GLM	−1.2	0.89	86	947	0.92	
	pGLS	−1.2	0.89	86	947	0.92	0.11
M_Optic Tectum_ ~ M_Brain_	GLM	−1.53	1	71	284.5	0.8	
	pGLS	−1.48	0.88	73	152.1	0.68	0.92
M_Tegmentum_ ~ M_Brain_	GLM	−0.99	0.8	71	419.7	0.85	
	pGLS	−0.95	0.8	71	319	0.82	0.68
M_Cerebellum_ ~ M_Brain_	GLM	−0.85	1.05	86	2510	0.97	
	pGLS	−0.85	1.06	86	1941	0.96	0.94
M_Medulla_ ~ M_Brain_	GLM	−0.54	0.8	86	868	0.91	
	pGLS	−0.53	0.85	86	908.8	0.91	0.87

### Brain organization

**Figure 4:**
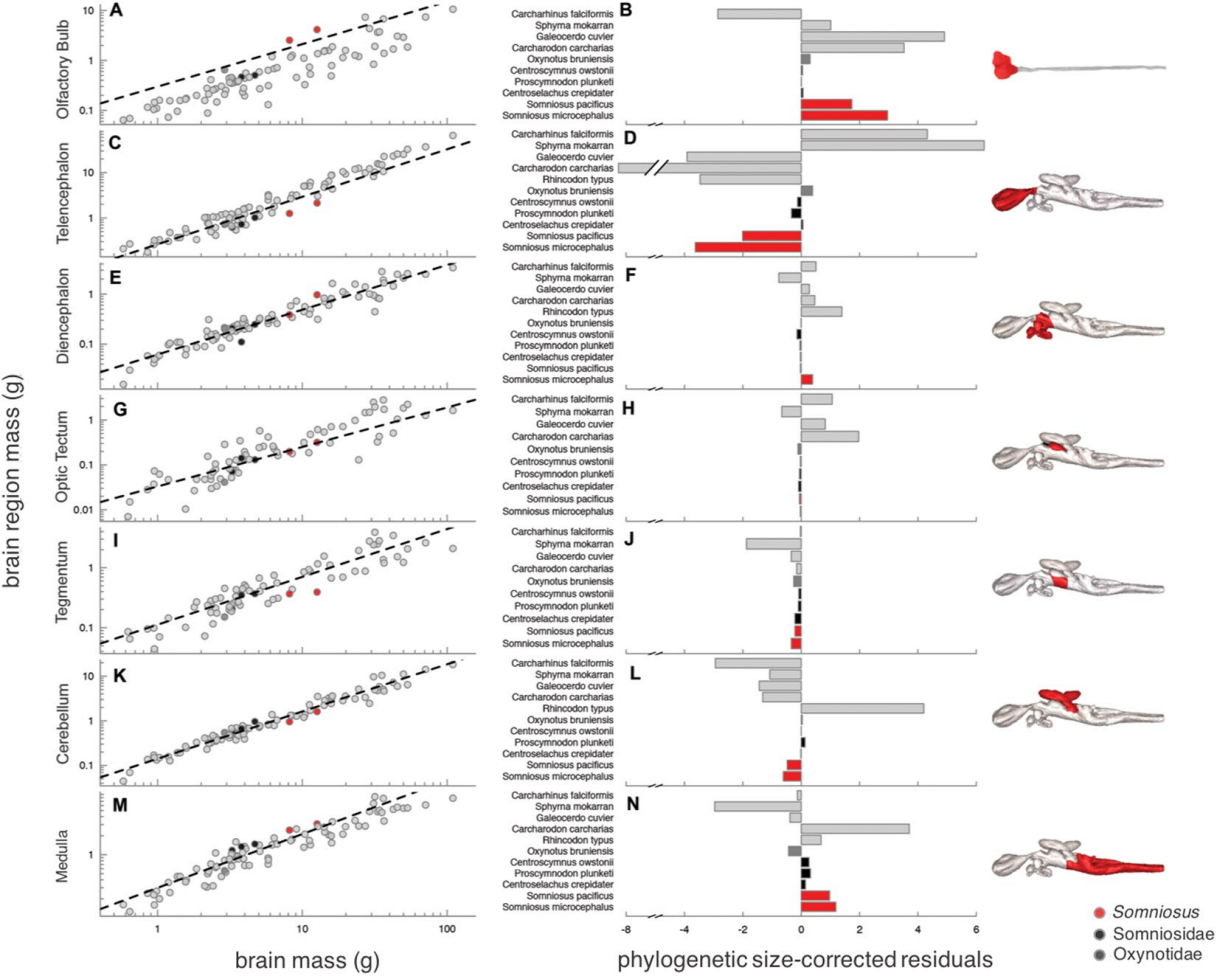
Phylogenetic generalized least squares (pGLS) regression of the (**A**) olfactory bulb mass (**C**) telencephalon mass, (**E**) diencephalon mass, (**G**) optic tectum mass, (**I**) tegmentum mass, (**K**) cerebellum mass, and (**M**) medulla mass with total brain mass across 84 species of cartilaginous fishes, indicating the relative size of major brain regions for *Somniosus microcephalus* and *S*. *pacificus* as compared to members of the Somniosidae (black circles), Oxynotidae (dark grey circles), and other species of sharks and holocephalans (light grey circles). Model results available in Table 2. Phylogenetically corrected residuals predicted from total brain mass for the (**B**) olfactory bulbs (**D**) telencephalon, (**F**) diencephalon, (**H**) optic tectum, (**J**) tegmentum, (**L**) cerebellum, and (**N**) medulla are presented for *Somniosus microcephalus*, *S*. *pacificus*, and nine other species for comparison.

The relative development of the major brain regions (olfactory bulbs, telencephalon, diencephalon, optic tectum, tegmentum, cerebellum, and medulla oblongata), expressed as phylogenetically corrected residuals (denoted as R_structure_; see Table 1), was determined for *S*. *microcephalus* and *S*. *pacificus* as compared to 84 other species (Fig. 4). Olfactory bulbs (OBs) scale with negative allometry (pGLS, α = 0.97, r^2^ = 0.80, p < 0.001, n = 83, Fig. 4A, Table 2) across this dataset. Within the family Somniosidae, the OBs comprise between 11% and 33% of the brain, with a clear hypertrophy of this structure in both *S*. *microcephalus* (R_OB_ = 2.96) and *S*. *pacificus* (R_OB_ = 1.74) (Fig. 4B, Table 1) compared with all other species examined. Other close relatives showed only moderate enlargement of this structure, with residuals ranging from R_OB_ = 0.01 (longnose velvet dogfish, *Centroselachus crepidater*) to R_OB_ = 0.31 (prickly dogfish, *Oxynotus bruniensis*) (Fig. 3B). *S*. *microcephalus* has one of the largest OBs of any species of cartilaginous fish described to date (Fig. 4B). The telencephalon (pGLS, α = 1.04, r^2^ = 0.96, p < 0.001, n = 84, Fig. 4C, Table 2) and diencephalon (pGLS, α = 0.89, r^2^ = 0.92, p < 0.001, n = 84, Fig. 4E, Table 2) are highly predictable from overall brain size, with the telencephalon scaling with positive allometry. The telencephalon of *S*. *microcephalus* (R_T_ = −3.59) and *S*. *pacificus* (R_T_ = −1.99) accounts for only 17% and 16% of the brain, respectively, showing relative reduction of this structure compared to other sharks (average = 36.3%^3^). Other members of the Somniosidae, such as *Centroscymnus owstonii* and *Centroselachus crepidater*, show similar reduction in telencephalon size (Fig. 4F). The diencephalon is relatively enlarged in *S*. *microcephalus* (R_D_ = 0.39) and average to reduced in *S*. *pacificus* (R_D_ = −0.03).

Scaling of the midbrain structures shows negative allometry for the optic tectum (pGLS, α = 0.87, r^2^ = 0.68, p < 0.001, n = 69, Fig. 4G, Table 2) and tegmentum (pGLS, α = 0.79, r^2^ = 0.82, p < 0.001, n = 69, Fig. 4I, Table 2). Midbrain development in the Greenland and Pacific sleeper sharks closely matches that of other somniosids, which possess a relatively average to reduced optic tectum [R_OT_ = −0.04 (*S*. *microcephalus*) and R_OT_ = −0.07 (*S*. *pacificus*), Fig. 4H] and a relatively average to reduced tegmentum [R_Tg_ = −0.34 (*S*. *microcephalus*) and R_Tg_ = −0.22 (*S*. *pacificus*), Fig. 4J]. The optic tectum comprises only ~2.5% of the brain in both species, a characteristic which is stereotypical of deep-sea dwelling somniosids (Fig. 4H).

**Figure 5:**
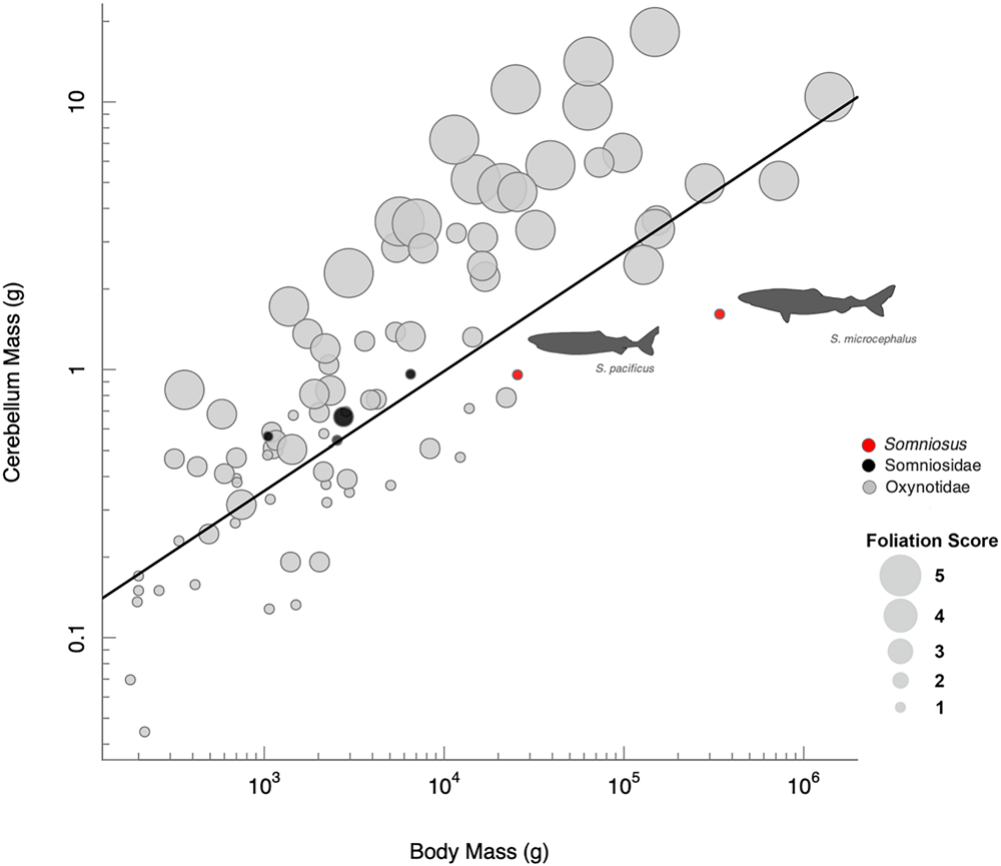
Phylogenetic generalized least squares (pGLS) regression of the cerebellum with body mass across 84 species of cartilaginous fishes. Circle size indicates foliation index score (1–5), with 1 corresponding to a smooth cerebellum surface and 5 indicating deep branch convexities and folds.

Hindbrain development is notable in these two species of sharks, particularly in relation to the cerebellum. The cerebellum scales with positive allometry against brain size (pGLS, α = 1.06, r^2^ = 0.96, p < 0.001, n = 84, Fig. 4K, Table 2) and both *S*. *microcephalus* (R_C_ = −0.61) and *S*. *pacificus* (R_C_ = −0.47) have a relatively reduced cerebellum, both in comparison to closely-related shark species and across cartilaginous fishes (Fig. 4L). Although the cerebellum can occupy over 30% of the brain in other sharks (e.g. *Rhincodon*, Fig. 4L), this brain region comprises less than 13% of the brain in both *S*. *microcephalus* and *S*. *pacificus*, and occupies between 17% and 20% of the brain in other somniosids^10^. According to the foliation index scheme developed by Yopak *et al*.^30^, both *S*. *microcephalus* and *S*. *pacificus* have a foliation index score of 1 (Table 1), which corresponds to a smooth cerebellar surface, without any invaginations or folds, similar to other somniosids. An increase in foliation is correlated with an increase in cerebellum size (pGLS, α = 1.50, r^2^ = 0.56, p < 0.001, n = 84), brain size (pGLS, α = 1.54, r^2^ = 0.52, p < 0.001, n = 84), and body size (pGLS, α = 0.56, r^2^ = 0.24, p < 0.01, n = 84) across this group; however, *Somniosus spp*. do not conform to this pattern (Fig. 5). Modeling of foliation index score versus cerebellum size, brain size, and body size showed that these factors exerted significant influence on cerebellar foliation. When all candidate models were assessed, the global model (model 4: foliation index score ~ cerebellum mass + brain mass + body mass) was the best predictor of foliation index score, although cerebellum mass alone (model 3: foliation index score ~ cerebellum mass) also has substantial support (ΔAIC = 0.44, Table S3).

The medulla scales with negative allometry against brain size (pGLS, α = 0.85, r^2^ = 0.91, p < 0.001, n = 84, Fig. 4M). The medulla of *S*. *microcephalus* (R_M_ = 1.21) and *S*. *pacificus* (R_M_ = 1.00) (Fig. 4N), are average to enlarged, comprising 24% and 30% of the brain, respectively. In general, other somniosids and oxynotids have relatively large medullas in comparison to other cartilaginous fishes, occupying between 17% and 30% of total brain mass.

## Discussion

Brain size and brain morphology may provide insights into shark behavior, particularly in species that are often difficult to study behaviorally due to their rarity and/or the extreme habitat in which they live. Of high ecological importance, the Greenland (*Somniosus microcephalus*) and Pacific sleeper (*S*. *pacificus*) sharks are the only non-lamnid shark species found in the Arctic, with a high prevalence of pinnipeds as a key component of their diet. In this study, the brain of these two unique species was examined as a way of predicting the relative importance of different sensory modalities. These sharks have a reduction in overall brain size and a marked reduction in the size of brain regions associated with higher cognitive functions, such as spatial learning and memory (e.g. the telencephalon^77^). They also possess relative reductions of the regions that receive the majority of afferents arising from the retinal ganglion cells (optic tectum) and one of the largest olfactory bulbs of any species described to date (comprising >30% of the brain), suggestive of a more olfactory-mediated, rather than a visually-mediated lifestyle.

As proposed by Jerison^11^, selection for larger brains (and/or enlargement of a particular brain region) are predicted to afford a functional advantage. However, little experimental evidence exists to empirically demonstrate this^12,78,79^. Thus, comparative studies of brain size and brain organization make the key assumption that there are correlations between brain regions and the functions or behaviors those regions modulate. However, correlatory evidence does not necessary reflect a causal relationship. It is important to note that this study is not a functional analysis; rather, it represents an attempt to use patterns of brain organization as a framework for exploring the relative importance of different sensory systems in poorly understood shark species. The extent to which morphological variation in the brain can directly confer differences in functional performance is of critical importance, but requires further study.

Within the cartilaginous fishes examined to date, *S*. *microcephalus* had a smaller than expected brain for its large body size (R_br_ = −1.54; Fig. 3). The only other published account of brain size in *S*. *microcephalus* documented a specimen of 280 kg body mass that had a brain size of 10.29g^71^, which is in line with allometric expectations for the *S*. *microcephalus* specimens examined in this study. A relatively small brain is a common characteristic for other deep-sea shark species^10^, including other members of Somniosidae (Fig. 3B). As brain tissue is energetically costly, a relatively small brain may reflect its lower metabolic rate^80,81^.

Although the degree to which brain size reflects enhanced cognitive capabilities continues to be highly contentious^82,83^, it is a common suggestion that encephalization informs behavioral complexity to some degree^84^. Of the few studies to date to explore a direct link between brain size and a cognitive task, guppies (*Poecilia reticulata*) selected for a 10% increase in brain size outperform smaller-brained individuals in cognitive learning tasks, which suggests a larger brain confers some cognitive advantages^12,79^, with tradeoffs between these cognitive benefits and energetic costs^80^. Although not empirically shown, reductions in brain size in cartilaginous fishes have been attributed to a number of factors, including more opportunistic, passive predation strategies^85,86^, a close association with the substrate^30,75^, and lower activity levels (reviewed in^3^). These behaviors have been suggested to lend themselves to sensory and motor requirements that are likely less cognitively demanding, as compared to agile hunters that occupy more spatially complex habitats, such as coral reefs^26,27,30^. Across other vertebrate groups, species with larger brains similarly tend to exhibit behavioral innovations in the form of increased sociality, complex learning, tool use, and foraging ecology, than do animals with small brains^e.g.^^17,21,78,87,88^.

In addition to a low degree of encephalization, the brain of *S*. *microcephalus* occupies only a small proportion of available endocranial space. A relatively small brain is a common attribute of many mature large-bodied shark species, such as *Carcharodon carcharias*, *Rhincodon typus*, and *Cetorhinus maximus*^30,86,89^, with a correspondingly small brain-to-endocranial-volume ratio^86,89–91^. The low levels of encephalization in large-bodied sharks may reflect an evolutionary increase in body growth without a concurrent increase in brain size, as opposed to an absolute reduction in brain size (termed “gigantism”^84^). However, small brains housed within expansive crania are not a consistent characteristic across all large-bodied cartilaginous fishes. Some of the most encephalized species, such as the great hammerhead, *Sphyrna mokarran*^30^ and mobulid rays^3,74^, can grow up to body sizes of 6 m in total length (*Sphyrna*)^43^ or >7 m in disc width (*Manta birostris*^92^) and possess brains that are either tightly housed in the chondrocranium (*Sphyrna*; Yopak, *pers*. *obs*) or are situated within an expansive cranial cavity (*Manta*^74^).

Encephalization is also correlated with a high degree of maternal investment in cartilaginous fishes, which exhibit the most diverse array of reproductive strategies of any vertebrate group^93^. These strategies range from egg-laying (lecithotrophy), with no investment beyond the yolk sac, to live-bearing (matrotrophy), where the developing embryo receives additional provisioning from the mother. Matrotrophic cartilaginous fishes have brains that are 20–70% larger than lecithotrophic species^28^, where the increased provisioning from the mother may provide a developing embryo with a cognitive advantage at birth. Although our knowledge of reproduction in *Somniosus* spp is limited, they are known to produce a high number of follicles^52,94^, although litter sizes are relatively low in both species examined here, with approximately 8–10 pups per litter^44^. *S*. *microcephalus* and *S*. *pacificus* are believed to be lecithotrophic live-bearers^46,52^, where embryos are nourished via a yolk-sac and pups are live^19^. This low reproductive output and lack of additional provisioning from mother to offspring may explain the low degree of encephalization in *Somniosus*, but more work is required on the reproductive biology of these sharks.

Unlike *S*. *microcephalus*, the brain of the *S*. *pacificus* examined in this study is relatively large in comparison to other somniosids and oxynotids and average-sized in comparison to all cartilaginous fishes examined (R_br_ = 1.86; Fig. 3). Although maturity was not assessed directly in *S*. *pacificus*, its body size (1.38 m TL; 25.6 kg) suggests this animal was a juvenile, as documented studies on the reproductive organs assert that *S*. *pacificus* matures at 3.65 m TL^52^. Cartilaginous fishes (as well as jawless and bony fishes, some amphibians and reptiles) experience indeterminate growth^95^. As such, their brains continue to grow throughout their lifespan, unlike other vertebrates that experience very little adult neurogenesis^96^. Although the brain grows through adulthood, the steepest period of growth is often during the early juvenile stages^97,98^, as seen in some jawless^99^ and bony fishes^100,101^ and reptiles^102^. Since the brain from an adult specimen of *S*. *pacificus* was not available for this study for comparison, the degree of encephalization documented in this study for *S*. *pacificus* may not be representative of the adult condition.

If natural selection is acting on a particular behavior or sensory characteristic of a species, there may be selective pressures similarly acting on the neural substratum that modulates that modality^15,16^. Previous studies on other members of Somniosidae have shown that they possess relatively small brains, with small telencephalons, small, smooth cerebellums^10,30^, and relatively large olfactory bulbs^29^ and medullas^10,70^. Like other somniosids, both *S*. *microcephalus* and *S*. *pacificus* possess a relatively small telencephalon and a relatively large medulla, which occupies between 17% (*S*. *microcephalus*) and 15% (*S*. *pacificus*) of the brain. *S*. *microcephalus* and *S*. *pacificus* have among the smallest telencephalons of any species described to date, including other deep-sea sharks and chimaerids^10^.

The telencephalon is implicated in multimodal sensory integration, complex behavioral control^75,103,104^, and higher cognitive functions, including allocentric place learning, avoidance learning, and long term memory^77,105–107^, as in teleosts^108^. In cartilaginous fishes, the telencephalon can comprise from 15–20% of the brain (e.g. in *Harriotta raleighana* and *Deania calcea*^10^) up to as much as 67% of the brain in *Sphryna mokarran*^30^. Given this variability, it has been proposed that telencephalon size may be indicative of behavioral complexity, whereby enlargement of this region is documented in shark species with increased sociality, strategic hunting, and navigating in spatially complex habitats^3,10,27,30,74^. The telencephalon is also one of the few brain regions to scale with positive allometry with brain size (Fig. 4E), such that larger brains become disproportionately composed of this structure, as similarly documented in mammals^26^. Functionally, lesions to various regions of the telencephalon, specifically the pallium, in some shark species have been shown to impair different types of learning^105–107^, which supports the assertion that the size of this structure likely reflects spatial learning and memory capabilities^77^. A small telencephalon in *Somniosus* is in line with phylogenetic expectations for this group and is also consistent with relative reduction of this structure in solitary species that do not dwell in spatially complex habitats^30^.

Olfaction is a critical sense in the aquatic realm and the detection of dissolved odorants is believed to mediate tasks ranging from predator avoidance, prey detection, and chemosensory communication with conspecifics (reviewed in^2,109^). Histological analysis of the peripheral system supports a well-developed olfactory capability in *S*. *microcephalus*, with a relatively high lamellar surface area and a high rate of renewal of olfactory receptor neurons (ORNs) in the olfactory epithelium^72,73^. The olfactory bulbs (OBs) receive primary projections from the ORNs and are associated with processing olfactory information^33,38^. Given the important role of olfaction in feeding, whereby many species follow odor plumes to their prey^1,2^, there may be selection pressures on the olfactory system for some species in this context. Both *S*. *microcephalus* and *S*. *pacificus* possess relatively large OBs (Fig. 1A,B), comprising 33% and 31% of the brain, respectively. In particular, compared across a range of other cartilaginous fishes, the OBs of *S*. *microcephalus* are among the largest of any species described to date (R_OB_ = 2.96), rivalled only by the tiger shark, *Galeocerdo cuvier* (R_OB_ = 4.89) and white shark, *Carcharodon carcharias* (R_OB_ = 3.50) (Fig. 4B), both large-bodied, highly migratory predators. The OBs can vary considerably in size and morphology across species^29,110^ and show a high degree of statistical independence from the rest of the brain^26,29^, a pattern also documented across other species, including bony fishes, amphibians, birds and mammals^26,111–113^. Correlations between OB variability and ecology has been widely used to confer olfactory capability in vertebrates^114–116^, although uncertainties exist regarding whether peripheral and central organization reflects functional specialization in cartilaginous fishes^38^. Variation in OB size may not be indicative of functional variability, and instead may reflect a tighter coupling between other, more highly-interconnected, regions of the brain^117^.

Previous work has shown relatively large OBs in shark species living in conditions where the use of visual information is in some way compromised, such as the deep-sea, or in species (e.g. *G*. *cuvier* and *C*. *carcharias*) that may follow chemosensory cues over long distances to locate cetacean carcasses^29,97,118^. Like *G*. *cuvier* and *C*. *carcharias*, *S*. *microcephalus* and *S*. *pacificus* also have a high prevalence of marine mammals in their diet^50,52,61,62,65^. Pinnipeds can create considerable odoriferous material and the resultant odor trails can likely be tracked over considerable distances by large marine predators^119,120^. In this context, a well-developed olfactory system would be evolutionarily advantageous in the Greenland and Pacific sleeper sharks, who may be relying on chemoreceptive cues to prey on pinnipeds.

In addition to foraging and locating conspecifics, olfaction has been proposed to play a role in linking locations in olfactory space for both short and long-distance navigation^121^. Accordingly, Jacobs^121^ proposes OB size should co-vary with navigational demand^29^, supported by enlarged OBs documented in migratory birds^122^, bats with enlarged wingspans^18^, and mammals with large home ranges^123^. Although there is not a large enough dataset to test this hypothesis directly in sharks^29^, the largest OBs are, in fact, found in highly migratory sharks, including *G*. *cuvier* and *C*. *carcharias* (Fig. 4). Similarly, both *S*. *microcephalus* and *S*. *pacificus* occupy a broad depth niche from the surface down to 2200 m, and can make daily vertical migrations, in addition to long-range latitudinal movements^48,49,58,124^. Should a function of the olfactory system be to map patterns of odorants in olfactory space, a correspondingly large OB might support this behavior in somniosids.

The optic tectum in *S*. *microcephalus* and *S*. *pacificus* is notably reduced, and occupies ~2.5% of the brain; this relative reduction in tectum size is characteristic for deep-sea dwelling somniosids (Fig. 4H). The superficial layers of the optic tectum receive the majority of primary projections arising from the retinal ganglion cells and are associated with visual processing^125–127^, in addition to receiving projections from other sensory modalities^128^. Despite its role as a multimodal integration center, variability in the size of the optic tectum is suggested to reflect visual specialization in non-mammalian species^8,69,88^ and often scales with a number of other aspects of the peripheral nervous system in fishes, including eye size, retinal area, the number of retinal ganglion cells and optic nerve axons, and overall retinal area^88,129,130^. In addition to living in low-light environments, among the most distinctive characteristic of *S*. *microcephalus* and *S*. *pacificus* is the presence of ocular lesions, generated by the large ectoparasitic copepod *Ommatokoita elongata*, which attaches to the cornea^54–56^. This parasite has a very high rate of occurrence in the Arctic sleeper sharks; it is documented in the majority of specimens of *S*. *microcephalus* caught in East Greenland, Baffin Island^58^, Cumberland Sound, and Svalbard, Norway^46^. Histological assessment of the attachment of these parasites (and subsequent larval deposition and/or infection) shows corneal lacerations, damage, and visual impairment^54,56^. Although whether *O*. *elongata* fully blinds its host is uncertain, these animals may only be capable of light/dark discrimination^56^. Despite the potential visual impairment caused by *O*. *elongata* and a relatively small eye^131^, *Somniosus spp*. clearly remain capable of capturing active prey, including pinnipeds^62,68^. Previous work has proposed these sharks depend primarily on chemoreception to find prey from a distance and only use motion detection and visual cues at close range^56^. Patterns of brain morphology in *S*. *microcephalus* and *S*. *pacificus*, with relatively large olfactory bulbs and relatively reduced optic tectums, similarly support an olfactory-mediated rather than a visually-mediated lifestyle. However, experimental evidence on both the visual and olfactory system is required to confirm this.

Cerebellar development in S. *microcephalus* and *S*. *pacificus* is noteworthy. Although the function of the cerebellum has been an area of considerable speculation throughout gnathostomes^132^, it is generally agreed that the cerebellum regulates motor control and motor learning^133^. Morphologically, the corpus cerebellum varies substantially in size, level of convolution (a.k.a. foliation) and symmetry in cartilaginous fishes^27,30,74,134–136^, and it has been suggested that this may reflect performance differences in cerebellar-dependent functions and behaviors^132^. Both *S*. *microcephalus* and *S*. *pacificus* possess a relatively small, smooth corpus, with low levels of cerebellar foliation - a pattern consistent with that previously described for squalomorph sharks^30,75^. In contrast, high levels of foliation have been documented in active, agile predators, such as *C*. *carcharias*^30^, species with extreme motor specializations, such as *Alopias spp*., which uses a rapid strike of the upper lobe of the caudal fin^30,118^, or large-bodied species that make long-distance migrations, such as *R*. *typus* and *C*. *maximus*^86,89^. In contrast, low levels of foliation are common in small, benthic species that rest on the seafloor or benthopelagic species that inhabit the deep-sea, suggestive of lower activity levels^10,30^. Foliation has been proposed to be an identifying feature of highly encephalized brains^26^ and that species with larger, more complex cerebella might have the ability to perform more multi-faceted motor tasks than their close relatives lacking these convolutions^137^. Although the exact mechanism for this characteristic is unknown in sharks, cerebellar foliation likely allows for an increased cerebellar surface area, while reducing the length of neuronal connections^26^, which may provide an energetically efficient means of coordinating larger body sizes or may serve to improve motor agility at the central level.

To our knowledge, *Somniosus spp*. are the only large-bodied (>3 m TL) shark species examined to date that have a smooth cerebellar corpus. An increase in foliation is correlated with an increase in cerebellum size, brain size, and body size (Table S3) across this group, where *S*. *microcephalus*, in particular, showed a marked deviation from expectation (Fig. 5). The lack of cerebellar complexity may reflect a reduction in fine-tuned motor behavior^26,30^, as Greenland sharks exhibit the slowest swimming speed for their body size compared to other representative cartilaginous fishes^59^. Tail beat frequency data also suggests a sluggish cruising speed of between 0.22 and 0.34 m/s^58,59^ and a maximum speed of 0.74 m/s^59^ for *S*. *microcephalus*, which is much slower than those estimated for species such as *C*. *carcharias*^120,138^ and *G*. *cuvier*^139,140^. Importantly, acceleration during burst swimming speeds for *S*. *microcephalus* (0.008 m/s^2^) are much lower than those recorded in pinnipeds^59^, which makes this species unlikely to achieve the high swimming speeds necessary for active pursuit of seals.

Ferrando *et al*.^72,73^ have proposed that *S*. *microcephalus* has well-developed olfactory capabilities and might rely heavily on chemoreception during foraging, which would include predating on marine mammals. Although it has been considered whether seals are being fed upon as carrion, characteristic corkscrew wounds found on living or deceased stranded animals suggest these pinnipeds are still being bitten while alive in the water^52,61,62,65^. Whole, intact small seals that appeared to be healthy at the time of ingestion also support the assertion that the seals were taken live^65^. Previous studies speculated that *S*. *microcephalus* may actively feed at ice holes (e.g. on over wintering *Delphinapterus leucas*^46^) and could be attracted to seal ice holes via a suite of olfactory, acoustic and visual cues, possibly using stealth and camouflage to approach and capture seals at the surface^58^. Other research suggest that *Somniosus* sp. may ambush sleeping seals^59,68^. Arctic seals often sleep in water, either underwater or at the surface^141,142^. It has been hypothesized that this sleeping behavior allows them to avoid predation by polar bears (*Ursus maritimus*) that feed on seals primarily on the sea ice^59,68^. However, unlike cetaceans or otariids, who employ unique unihemispheric sleep patterns as a means to remain active while one half of the brain is in a sleeping state^143,144^, phocid seals exhibit bilaterally symmetrical (bihemispheric) sleep patterns, characteristic of terrestrial mammals^141^. Although protected from being hunted on the ice by *U*. *maritimus*, an immobilized state during sleep, may leave Arctic seals vulnerable to cryptic predators in the water, including *Somniosus*.

## Conclusions

Although this was not a functional analysis, this study asserts that brain size and organization may provide insight into sensory specialization, particularly in data-deficient species that are often difficult to study behaviorally. The Greenland (*S*. *microcephalus*) and Pacific sleeper (*S*. *pacificus*) sharks are the only large-bodied predatory shark species to occur in the Arctic. In addition to occurring at depths up to 2200 m, these sharks often feed under the ice with a high prevalence of marine mammals, particularly pinnipeds, found in their stomachs, despite a degree of parasite-induced visual impairment. Although similar in many respects to other deep-sea squaliform sharks, patterns of brain morphology in the Greenland and Pacific sleeper sharks show unique characteristics. These sharks have a relatively reduced optic tectum and one of the largest olfactory bulbs of any species described to date, suggestive of an increased reliance on olfaction over vision. In addition, a notable characteristic of the brain of *Somniosus* is a small, smooth cerebellar corpus, the first to be documented in a large-bodied (>3 m) shark species. Taken together, brain morphology suggests a slow-moving predator with well-developed olfactory capabilities.

## Methods

### Animals

Brains from three specimens of *Somniosus microcephalus* (1 male, 2 female) were collected by the Norwegian Polar Institute (Table S1) in accordance with the ethical guidelines of the University of Windsor and the Norwegian Research Council. This project (Animal Utilization Project Proposal #07-11) was approved by the University of Windsor Animal Care committee, whose policies and procedures are designed to comply with those of the Canadian Council on Animal Care. Similarly, the project activities in Norway (within project 184644/S40/mo) of the Norwegian Research Council followed the rules and regulations of the national animal research ethics committee, under permits from the Governor of Svalbard. One specimen of *S*. *pacificus* (female) was collected on western side of Prince William Sound by K.J. Goldman of the Alaska Department of Fish and Game (ADF&G). This specimen was acquired as an incidental bycatch mortality during a routine trawl survey by ADF&G, and the brain donated for use in this study (Table S1).

Morphometrics (body mass, TL, FL, PCL) were collected for all specimens, where possible (Table S1). For *S*. *pacificus*, body mass was estimated based on published length-weight relationships^47^. Although *S*. *microcephalus* is predicted to mature at *c*. 3.0 m TL^46,52^, maturity was assessed in all specimens and none were mature. Maturity for *S*. *pacificus* was predicted based on published estimates for length-at-maturity values of *c*. 3.65 m TL^52,145^, although these are highly variable. Using these data, animals were assumed to be immature (*S*. *pacificus*) or sub-adult (*S*. *microcephalus*) (Table S1).

### Tissue processing

Brains were exposed within the cranium and tissue was preserved in an aldehyde-based fixative (10% formalin in 0.1 M phosphate buffer). Following a period of fixation, brains were excised from the cranium and, following removal of the meninges, blood vessels, choroid plexa, connective tissue, and the cranial nerves, the brains were blotted and weighed to the nearest 0.01 g. Olfactory bulbs were separated from the peduncles and weighed. Brain mass was not corrected for fixation. Body mass information was recorded (or estimated from length-weight relationships) on fresh, unfixed samples after Yopak *et al*.^30^.

### Imaging and segmentation methods

Given the rarity of these samples, brain organization was assessed non-invasively using magnetic resonance imaging (MRI). Following fixation, brains were transferred to 0.1M PB + 0.01% sodium azide for at least 14 days to remove excess fixative before transferring to fresh 0.1M PB + 0.01% sodium azide with the addition of 5 mM of the contrast agent Dotarem (for up to 1 week at 4 °C). MR image data was acquired from contrast-enhanced, fixed brains.

Brains were removed from the contrast agent solution and embedded in an inert imaging media (Fluorinert FC770). Imaging was performed on a Bruker BioSpec 94/30 US/R (9.4 T) small animal scanner, with an AVANCE III HD console, and using BGA-12SHP gradients (max 0.66 T m^−1^), a 72 mm ID H-1 quadrature volume RF coil, and ParaVision 6.0 software at the Center for Microscopy, Characterization and Analysis at The University of Western Australia. High-resolution (100 μm isotropic), T1-weighted images were acquired using a 3D FLASH sequence with RF spoiling. Images were produced with high contrast between gray and white matter in cartilaginous fish brains^86,136^, from which structural characteristics were derived (Fig. 2A,B). The pulse sequence parameters used for this study are shown in Table S2.

Three-dimensional data, acquired from high-resolution MRI, were digitally segmented using ITK-SNAP, a cross-platform, open-source application that includes a toolbox for manual delineation and a simple intuitive interface for user-guided automatic segmentation using an active contour (level set) algorithm^146^. Six major brain structures, the telencephalon, diencephalon, mesencephalon (sub-divided into the optic tectum and tegmentum), cerebellum, and medulla oblongata were individually segmented from the 3D data (Fig. 2C–F). Boundaries for these brain regions were identified using the criteria of Northcutt^75^, Yopak *et al*.^30^, and Yopak and Lisney^69^. The volume of each brain region as a proportion of the total brain volume was acquired from these digital segmentations and multiplied by total brain mass (less the olfactory bulbs) to acquire the mass of each structure, which was then compared across species.

The degree of folding of the cerebellum of *S*. *microcephalus* and *S*. *pacificus* was assessed using a visual grading index from Yopak *et al*.^30^. This method involved assigning a quantitative score (1–5) to the length, depth, and number of folds in the cerebellum (Table 1). A grade of 1 corresponded to a smooth cerebellar surface with no folding, increasing in complexity to a grade of 5, which corresponded to extreme foliation with deep, branched grooves^30^. Although not as quantitatively rigorous as other methods^136^, the visual foliation index score was available on the greatest number of species^10,27,74^, thereby facilitating a broad comparative analysis.

Brain mass and brain organization data from *S*. *microcephalus* and *S*. *pacificus* from this study were combined with comparable data on total brain mass (n = 117) and brain region mass (olfactory bulbs (n = 83); telencephalon, diencephalon, cerebellum, and medulla (n = 84)) and foliation index score (n = 84) from additional cartilaginous fish species^27,29,30,71,75^. A second dataset was compiled for published data on both optic tectum and tegmentum mass across 69 species^69^. Olfactory bulb data were not available for *Rhincodon typus*.

### Quantification and statistical analysis

The scaling relationship between brain and body size across cartilaginous fish species was examined using log_10_ transformed data and the allometric relationship was determined using generalized least-squares (GLS) regression. However, GLS regression does not account for the relatedness of species and treats species values as statistically independent data points, yet closely related species share many characters through common descent rather than through independent evolution^147^. The use of GLS regression without consideration of underlying phylogenetic relationships can result in overestimation of correlations and result in Type I errors^148^. Therefore, allometric relationships were also estimated using a phylogenetic generalized least-squares (pGLS) approach for both brain mass versus body mass and brain structure mass versus brain mass^149,150^. We used a maximum likelihood approach to simultaneously estimate the phylogenetic signal in the model parameters and error structure^149,151^ using Pagel’s λ statistic^150^. A λ value of one indicates a correlation between species reflecting Brownian motion, while a λ of zero indicates no correlation between species^150,152^. Results of the pGLS model are presented together with the results from GLS analyses (Table 2) for brain versus body mass to enable comparison with previous research.

The phylogeny for the taxa set was created by pruning a larger 610 species molecular tree^153^ to the desired taxa set (Supplementary Fig. S1). Using this reduced tree, pGLS models of evolutionary change were constructed, using the caper^154^ and nlme^155^ packages in R^156^. From each model (brain mass ~ body mass) or (brain structure mass ~ brain mass), standardized pGLS residuals, or vertical deviations from the predicted slope, were also calculated using the phytools packages^157^. These residuals reflect interspecific variation in the relative size of the brain and each major structure, independent of phylogenetic constraints^158,159^ (Supplementary Figure S2). Goodness-of-fit measures (r^2^) were calculated using caper, which is estimated by comparing the goodness-of-fit of the candidate model to an intercept only model^154^.

Factors shaping cerebellar foliation were examined using pGLS in a model selection framework. Four candidate models were constructed: (1) body mass, (2) brain mass, (3) cerebellum mass, (4) body mass, brain mass, and cerebellum mass. We used Akaike Information Criteria (AIC) to identify the models that best explain our data from the suite of candidate models^160,161^. Model significance was based on a ∆AIC of 2, with the model yielding the lowest AIC value being the most supported.

## Supplementary information

Supplementary Information

## Data Availability

The datasets generated and analyzed during the current study that have not already been provided in the accompanying tables are available from the corresponding author on reasonable request.
