# Supplementary material for: Comparative Brain Morphology of the Greenland and Pacific Sleeper Sharks and its Functional Implications
Source: Sci Rep. 2019 Jul 11;9:10022. doi: 10.1038/s41598-019-46225-5 (PMC6624305; doi:10.1038/s41598-019-46225-5)
Supplement: Supplementary file 1 — Supplementary Information [file 41598_2019_46225_MOESM1_ESM.pdf]

Title: Comparative Brain Morphology of the Greenland and Pacific Sleeper Sharks and its Functional Implications

Kara E. Yopak[1,*], Bailey C. McMeans[2], Christopher G. Mull[3], Kirk W. Feindel[4], Kit M. Kovacs[5], Christian Lydersen[5], Aaron T. Fisk[6] and Shaun P. Collin[7,8]

[1]Department of Biology and Marine Biology and the UNCW Center for Marine Science, University of North Carolina Wilmington, Wilmington, NC, 28403 United States

[2]Department of Biology, University of Toronto Mississauga, Mississauga, ON, L5L 1C6, Canada

[3]Earth to Ocean Research Group, Department of Biological Sciences, Simon Fraser University, Burnaby, BC, V5A 1S6, Canada

[4]Center for Microscopy Characterisation and Analysis, University of Western Australia, Crawley, WA 6009, Australia

[5]Norwegian Polar Institute, Fram Centre, N-9296 Tromsø, Norway

[6]Great Lakes Institute for Environmental Research, University of Windsor, N9B 3P4 Windsor, ON, Canada

[7]Oceans Graduate School and The Oceans Institute, The University of Western Australia, Crawley WA 6009, Australia.

[8]School of Life Sciences, La Trobe University, Bundoora 3086, Victoria, Australia.

*Correspondence: Kara E. Yopak, yopakk@uncw.edu

Supplemental Information

Supplementary Figure Legends

Figure S1. A phylogenetic tree of the 117 species used in this study, created by pruning a larger (610 species) molecular tree[1] to the desired taxa set. Species for which brain size data (black squares), brain organization data (blue squares), and data on sub-regions of the mesencephalon (i.e. optic tectum and tegmentum) (grey squares) are available as indicated. The species examined in this study (*Somniosus microcephalus* and *S. pacificus*) are outlined in red.

Figure S2. Scatterplot of the phylogenetically size-corrected residuals, predicted from total brain mass, for the (**A)** olfactory bulbs (**B**) telencephalon, (**C**) diencephalon, (**D**) optic tectum, (**E**) tegmentum, (**F**) cerebellum, and (**G**) medulla. The species examined in this study (*Somniosus microcephalus* and *S. pacificus*) are indicated (red), together with three other species from the Somniosidae (black) and one species from the Oxynotidae (dark grey) families for comparison. Data on most brain regions (**A-C**, **F-G**) is from a comparative dataset across 84 species of cartilaginous fishes, compiled from the literature[2-5]. Data on the optic tectum and tegmentum (**D, E**) is from a comparative dataset across 69 species[6].

Supplementary Tables

Table S1. Morphometrics (total length (TL), fork length (FL), precaudal length (PCL), and body mass) from the three individual specimens of the Greenland Shark (*Somniosus microcephalus*) and one specimen of the Pacific Sleeper Shark (*S. pacificus*), where available. *Body mass was calculated based on a published length-weight relationship[7].

Species	Specimen	Sex	TL (cm)	FL (cm)	PCL (cm)	Body Mass (kg)
Somniosus microcephalus	01	M	324	298	--	323
Somniosus microcephalus	02	F	296	282	--	347
Somniosus microcephalus	03	F	325	303	--	352
Somniosus pacificus	01	F	--	--	117.5	25.59*

Table S2. Pulse sequence parameters for the T1-weighted scans of the brains of *Somniosus microcephalus* (SM) and *S. pacificus* (SP) specimens, including echo time (TE), repetition time (TR), flip angle, field of view (FOV), matrix size, number of averages (NEX), and acquisition time.

Specimen	Pulse Sequence	TE (ms)	TR (ms)	Flip angle (deg)	FOV (mm)	Matrix Size	NEX	Acq time
SM01	FLASH 3D	7.5	25	15	87 mm x 22 mm x 15 mm	870 x 220 x 150	48	11h 0m
SM02	FLASH 3D	7.5	25	15	92 mm x 21 mm x 16 mm	920 x 210 x 160	48	11h 12m
SM03	FLASH 3D	7.5	25	15	92 mm x 20 mm x 17 mm	920 x 200 x 170	48	11h 20m
SP01	FLASH 3D	7.5	25	15	82 mm x 25 mm x 15 mm	820 x 250 x 150	48	12h 30m

Table S3. Candidate models shaping foliation index score were tested using pGLS in a model selection framework: (1) body mass, (2) brain mass, (3) cerebellum mass, (4) body mass, brain mass, and cerebellum mass. The best-fit model(*), as determined by AIC score (Model 4), is emboldened, although Model 3 (ΔAIC=0.44) also has substantial support.

**Model**		**d.f.**	**F-stat**	**r^2**	**AIC**	**Δ AIC**	**λ**
1	Foliation ~ M_{Body}	86	27.55	0.23	220.9	41	0.92
2	Foliation ~ M_{Brain}	86	91.13	0.51	187	7.1	0.86
3	Foliation ~ $M_{Cerebellum}$	86	107.1	0.55	180.34	0.44	0.85
**4**	**Foliation ~ M_{Body} + M_{Brain} + $M_{Cerebellum}$**	**84**	**38.17**	**0.56**	**179.9***	**0**	**0.85**

Supplementary References

1 Stein, R. *et al.* Global priorities for conserving the evolutionary history of sharks, rays, and chimaeras. *Nature Ecol. Evol.* **2**, 288-298 (2018).

2 Myagkov, N. A. The brain sizes of living Elasmobranchii as their organization level indicator. I. General Analysis. *J. Hinforsch.* **32**, 553-561 (1991).

3 Northcutt, R. G. in *Sensory Biology of Sharks, Skates, and Rays* (eds E.S. Hodgson & R.F. Mathewson) 117-194 (Office of Naval Research, 1978).

4 Yopak, K. E. Neuroecology in cartilaginous fishes: The functional implications of brain scaling. *J. Fish Biol.* **80**, 1968-2023 (2012).

5 Yopak, K. E., Lisney, T. J. & Collin, S. P. Not all sharks are "swimming noses": Variation in olfactory bulb size in cartilaginous fishes. *Brain Struc. Func.* **220**, 1127-11143 (2015).

6 Yopak, K. E. & Lisney, T. J. Allometric scaling of the optic tectum in cartilaginous fishes. *Brain Behav. Evol.* **80**, 108-126 (2012).

7 Orlov, A. & Baitalyuk, A. Spatial distribution and features of biology of Pacific sleeper shark *Somniosus pacificus* in the North Pacific. *J. Ichthy.* **54**, 526-546 (2014).

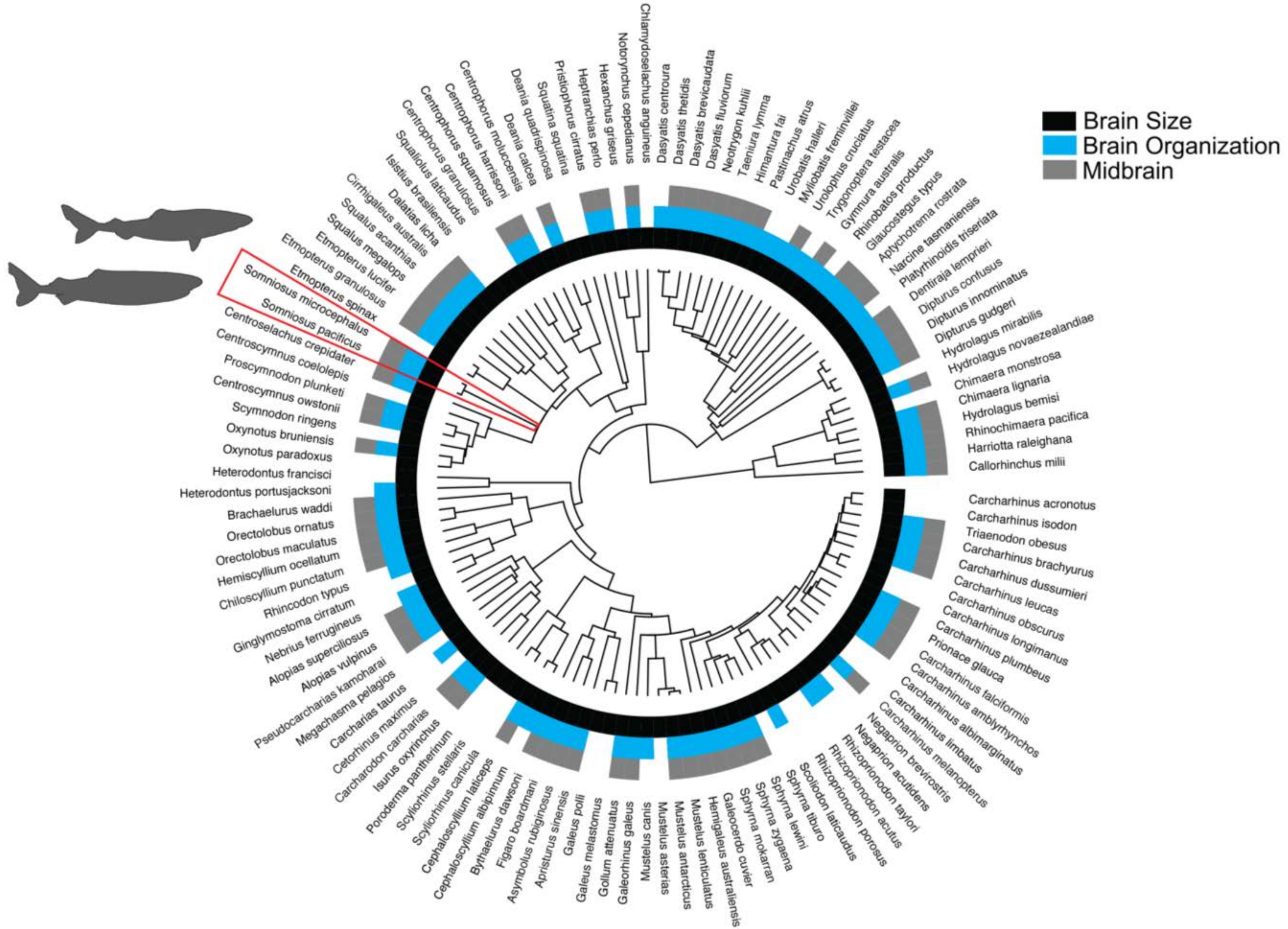

Brain Size
Brain Organization
Midbrain
Chlamydoselachus anguineus
Notorynchus cepedianus
Hexanchus griseus
Heptranchias perlo
Pristiophorus cirratus
Squatina squatina
Deania quadrispinosa
Deania calcea
Centrophorus moluccensis
Centrophorus harrissoni
Centrophorus squamosus
Centrophorus granulosus
Squaliolus laticaudus
Isistius brasiliensis
Dalatias licha
Cirrhigaleus australis
Squalus acanthias
Squalus megalops
Etmopterus lucifer
Etmopterus granulosus
Etmopterus spinax
Somniosus microcephalus
Somniosus pacificus
Centroselachus crepidater
Centroscymnus coelolepis
Proscymnodon plunketi
Centroscymnus owstonii
Scymnodon ringens
Oxynotus bruniensis
Oxynotus paradoxus
Heterodontus francisci
Heterodontus portusjacksoni
Brachaelurus waddi
Orectolobus ornatus
Orectolobus maculatus
Hemiscyllium ocellatum
Chiloscyllium punctatum
Rhincodon typus
Ginglymostoma cirratum
Nebrius ferrugineus
Alopias superciliosus
Alopias vulpinus
Pseudocarcharias kamoharai
Megachasma pelagios
Carcharias taurus
Cetorhinus maximus
Carcharodon carcharias
Isurus oxyrinchus
Poroderma pantherinum
Scyliorhinus stellaris
Scyliorhinus canicula
Cephaloscyllium laticeps
Cephaloscyllium albipinnum
Bythaelurus dawsoni
Figaro boardmani
Asymbolus rubiginosus
Apristurus sinensis
Galeus polli
Galeus melastomus
Gollum attenuatus
Galeorhinus galeus
Mustelus canis
Mustelus asterias
Mustelus antarcticus
Mustelus lenticulatus
Hemigaleus australiensis
Galeocerdo cuvier
Sphyrna mokarran
Sphyrna zygaena
Sphyrna lewini
Sphyrna tiburo
Scoliodon laticaudus
Rhizoprionodon porosus
Rhizoprionodon acutus
Rhizoprionodon taylori
Negaprion acutidens
Negaprion brevirostris
Carcharhinus melanopterus
Carcharhinus limbatus
Carcharhinus albimarginatus
Carcharhinus amblyrhynchos
Carcharhinus falciformis
Prionace glauca
Carcharhinus plumbeus
Carcharhinus longimanus
Carcharhinus obscurus
Carcharhinus leucas
Carcharhinus dussumieri
Carcharhinus brachyurus
Triaenodon obesus
Carcharhinus isodon
Carcharhinus acronotus
Callorhinchus milii
Harriotta raleighana
Rhinochimaera pacifica
Hydrolagus bemisi
Chimaera lignaria
Chimaera monstrosa
Hydrolagus novaezealandiae
Hydrolagus mirabilis
Dipturus gudgeri
Dipturus innominatus
Dipturus confusus
Dentiraja lemprieri
Platyrhinoidis triseriata
Narcine tasmaniensis
Aptychotrema rostrata
Glaucostegus typus
Rhinobatos productus
Gymnura australis
Trygonoptera testacea
Urolophus cruciatus
Myliobatis freminvillei
Urobatis halleri
Pastinachus atrus
Himantura fai
Taeniura lymma
Neotrygon kuhlii
Dasyatis fluviorum
Dasyatis brevicaudata
Dasyatis thetidis
Dasyatis centroura

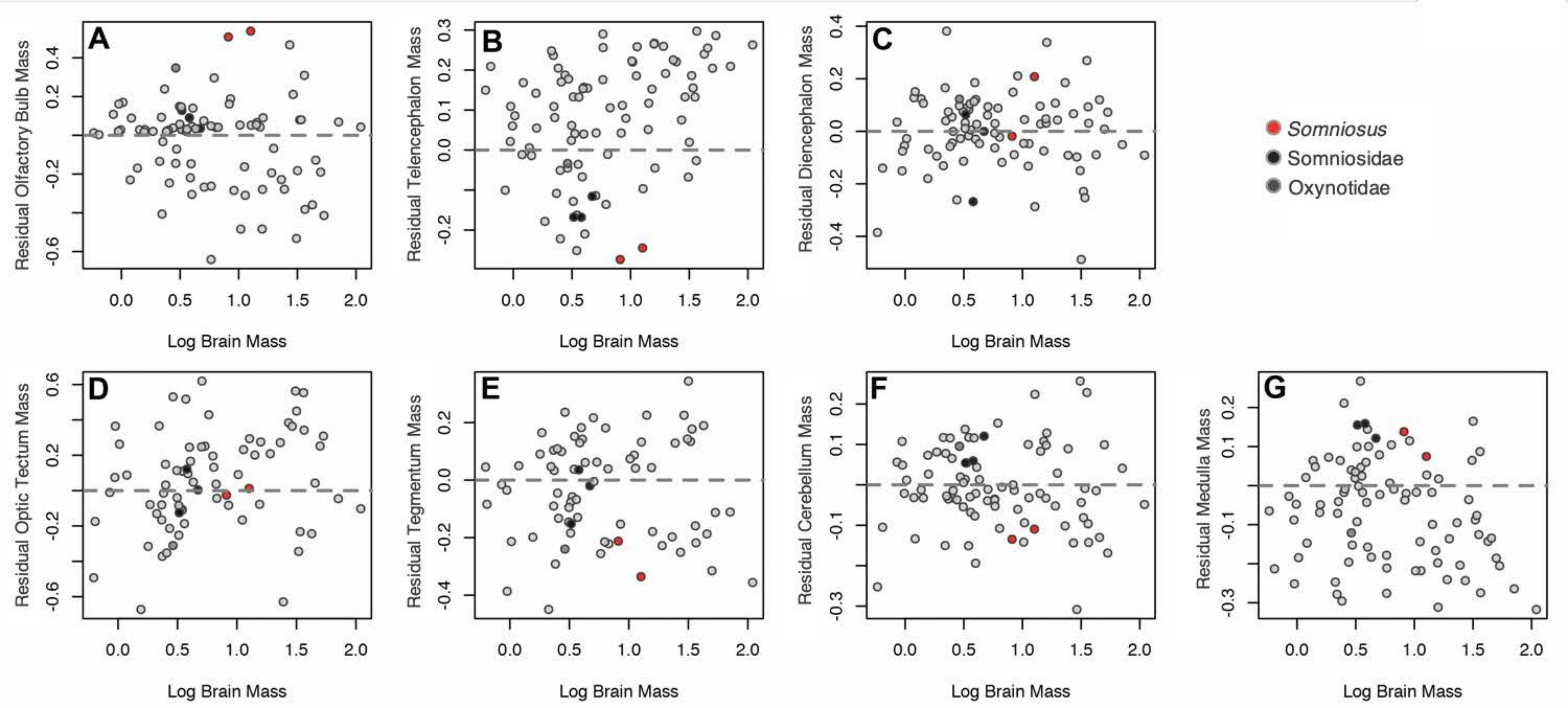
A
Residual Olfactory Bulb Mass
B
Residual Telencephalon Mass
C
Residual Diencephalon Mass
D
Residual Optic Tectum Mass
E
Residual Tegmentum Mass
F
Residual Cerebellum Mass
G
Residual Medulla Mass
Log Brain Mass
Somniosus
Somniosidae
Oxynotidae
